# Acute metabolic decompensation due to influenza in a mouse model of ornithine transcarbamylase deficiency

**DOI:** 10.1242/dmm.013003

**Published:** 2013-11-21

**Authors:** Peter J. McGuire, Tatiana N. Tarasenko, Tony Wang, Ezra Levy, Patricia M. Zerfas, Thomas Moran, Hye Seung Lee, Brian J. Bequette, George A. Diaz

**Affiliations:** 1 National Human Genome Research Institute, National Institutes of Health, Bethesda, MD 20892, USA.; 2 Department of Genetics & Genomic Sciences, Mount Sinai School of Medicine, New York, NY 10029, USA.; 3 Office of Research Services, Division of Veterinary Resources, National Institutes of Health, Bethesda, MD 20892-2324, USA.; 4 Department of Microbiology, Mount Sinai School of Medicine, New York, NY 10029, USA.; 5 Department of Pediatrics, University of South Florida, Tampa, FL 33620, USA.; 6 Department of Animal and Avian Sciences, University of Maryland, College Park, MD 20742-2311, USA.

**Keywords:** Urea cycle disorder, Hyperammonemia, Infection, Influenza

## Abstract

The urea cycle functions to incorporate ammonia, generated by normal metabolism, into urea. Urea cycle disorders (UCDs) are caused by loss of function in any of the enzymes responsible for ureagenesis, and are characterized by life-threatening episodes of acute metabolic decompensation with hyperammonemia (HA). A prospective analysis of interim HA events in a cohort of individuals with ornithine transcarbamylase (OTC) deficiency, the most common UCD, revealed that intercurrent infection was the most common precipitant of acute HA and was associated with markers of increased morbidity when compared with other precipitants. To further understand these clinical observations, we developed a model system of metabolic decompensation with HA triggered by viral infection (PR8 influenza) using *spf-ash* mice, a model of OTC deficiency. Both wild-type (WT) and *spf-ash* mice displayed similar cytokine profiles and lung viral titers in response to PR8 influenza infection. During infection, *spf-ash* mice displayed an increase in liver transaminases, suggesting a hepatic sensitivity to the inflammatory response and an altered hepatic immune response. Despite having no visible pathological changes by histology, WT and *spf-ash* mice had reduced CPS1 and OTC enzyme activities, and, unlike WT, *spf-ash* mice failed to increase ureagenesis. Depression of urea cycle function was seen in liver amino acid analysis, with reductions seen in aspartate, ornithine and arginine during infection. In conclusion, we developed a model system of acute metabolic decompensation due to infection in a mouse model of a UCD. In addition, we have identified metabolic perturbations during infection in the *spf-ash* mice, including a reduction of urea cycle intermediates. This model of acute metabolic decompensation with HA due to infection in UCD serves as a platform for exploring biochemical perturbations and the efficacy of treatments, and could be adapted to explore acute decompensation in other types of inborn errors of metabolism.

## INTRODUCTION

The urea cycle (UC; supplementary material Fig. S1) is present only in the liver and serves two purposes: (1) the *de novo* biosynthesis and degradation of arginine, and (2) the incorporation of excess nitrogen into urea. UC disorders (UCDs) are caused by loss of function in any of the enzymes responsible for ureagenesis, and are characterized by life-threatening episodes of acute metabolic decompensation with hyperammonemia (HA). The incidence of these disorders has been estimated at 1 in 30,000 live births (Summar and Tuchman, 2001). UCD can be considered in two groups. In proximal (mitochondrial) UCD [N-acetylglutamate synthetase (NAGS), carbamoyl phosphate synthetase 1 (CPS1) and ornithine transcarbamylase (OTC) deficiencies], ammonia disposal is severely compromised. In distal (cytosolic) UCD [arginosuccinate synthetase (ASS), arginosuccinate lyase (ASL) and arginase (ARG1) deficiencies], ammonia disposal is not as severely impaired and characteristic amino acid metabolites accumulate.

Acute metabolic decompensation with HA in UCDs is precipitated by dietary non-adherence, enhanced protein catabolism due to protein and/or caloric over-restriction, or intercurrent infection (Summar et al., 2008). Intercurrent infection, particularly with respiratory viruses, is the most common trigger of HA, accounting for >34% of episodes in a cohort of UCD subjects (Summar et al., 2008; Tuchman et al., 2008). Although it has not been systematically examined, intercurrent infection is suspected to result in greater morbidity versus other precipitants, based on anecdotal clinical experience (Singh et al., 2005). For UCDs, infection results in elevated plasma ammonia of longer duration when compared to other precipitants, resulting in prolonged hospital stays and increased utilization of medical resources. The current paradigm for acute HA treatment centers on addressing the increased whole-body protein catabolism brought on by protein and/or caloric insufficiency that can occur in dietary over-restriction or intercurrent infection. Reversing catabolism by drastically increasing parenteral caloric intake, regardless of the precipitant, has been the mainstay of acute HA crisis treatment (Singh et al., 2005). However, the pathophysiological processes behind different HA precipitants might be distinct, raising the possibility of targeted therapies that could alter the hospital course of UCD patients.

Given the well-recognized propensity of individuals with UCDs to experience severe and at times fatal HA, a translational approach was undertaken to develop a model of acute HA due to infection in UCD and describe the mechanisms of acute metabolic decompensation. To explore the etiology, clinical characteristics and morbidity associated with HA events, the longitudinal database of the Urea Cycle Disorders Consortium (UCDC) was queried to assess the severity of HA experienced by patients with the most common UCD, OTC deficiency. Infection was noted to be the most common precipitant of acute HA, with indicators of increased morbidity being present compared with other precipitants. To recapitulate a robust inflammatory response and to explore further perturbations in UC function as experienced by OTC patients during infection, an experimental model of infection-associated acute HA was created by inoculating *spf-ash* mice, a model of OTC deficiency, with the influenza A/Puerto Rico/8/34 (PR8) virus. Both wild-type (WT) and *spf-ash* displayed perturbations in UC enzyme activity and intermediates, suggesting that influenza infection can suppress a subset of hepatic functions, including enzymes that are already compromised in patients with UCD.

**TRANSLATIONAL IMPACT**

**Clinical issue**

The urea cycle functions to incorporate ammonia, generated by normal metabolism, into urea. Urea cycle disorders (UCDs), which fall under the category of inborn errors of metabolism, are caused by loss-of-function mutations in any of the enzymes responsible for ureagenesis, and are characterized by potentially life-threatening episodes of acute metabolic decompensation with hyperammonemia (HA). Acute HA in UCD can be precipitated by any factor that affects metabolic balance, such as: dietary indiscretion, enhanced protein catabolism due to dietary over-restriction, or infection. Intercurrent infection is the most common precipitant of acute HA, with respiratory viruses being a leading cause. The aim of this study was to explore perturbations in UC function as experienced by UCD patients during infection.

**Results**

In a prospective analysis of a cohort of patients with ornithine transcarbamylase deficiency (OTCD), the most common UCD, the authors identified infection as the most common identifiable cause of acute decompensation with HA, and this was associated with markers of increased morbidity. To further understand these clinical observations, the authors developed a model system of metabolic decompensation with HA triggered by viral infection [influenza A/Puerto Rico/8/34 (PR8) virus] using *spf-ash* mice, an animal model of OTCD. In response to infection with PR8, *spf-ash* mice displayed an altered hepatic immune response. Unlike wild-type (WT) mice, *spf-ash* mice also displayed elevated liver transaminases, suggesting increased hepatic sensitivity to infection. Despite having no visible pathological changes detectable by histology, WT and *spf-ash* mice showed reduced activities of the first two enzymes of the urea cycle: carbamoyl phosphate synthetase 1 and ornithine transcarbamylase. In addition to these enzyme perturbations, *spf-ash* mice showed increased hyperammonia and, in contrast to WT mice, failed to increase ureagenesis during infection. Liver amino acid analysis revealed further perturbations in urea cycle function, with reductions seen in the intermediates aspartate, ornithine and arginine during infection.

**Implications and future directions**

These results provide insights into the metabolic perturbations triggered by influenza infection in the presence of UCD, including a reduction in levels of urea cycle intermediates. The findings could be important for the development of new approaches for the management of acute HA in UCDs. Regardless of the nature of the acute HA precipitant, the current medical management strategy is the same: to abolish protein intake and supplement with high caloric intake. These measures are not always successful. The experimental model used here could provide a platform for evaluating the efficacy of urea cycle intermediates alone or in combination with immune modifiers. This model could also be adapted to explore acute decompensation and the efficacy of treatments in other types of inborn errors of metabolism.

## RESULTS

### Infection is associated with indicators of increased morbidity in individuals with OTC

**Fig. 1.**
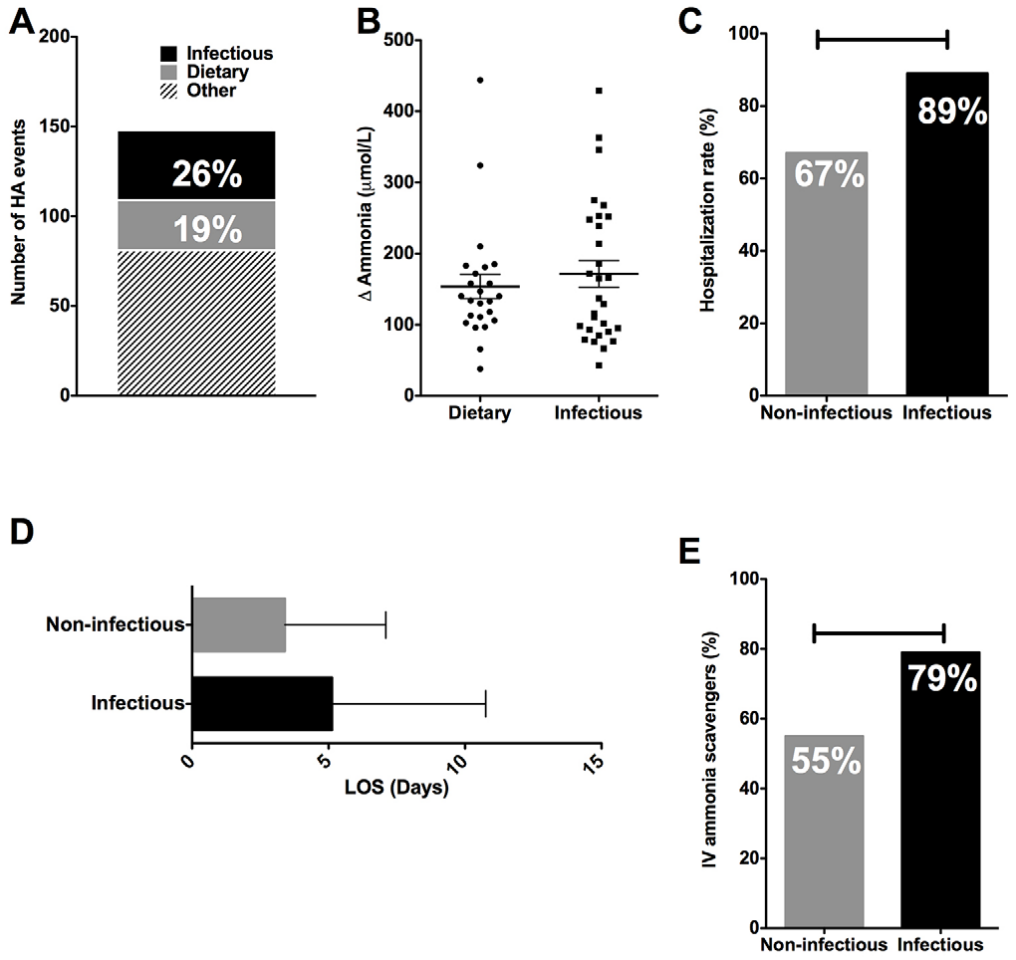
**Differences in clinical findings in patients with HA due to infectious or dietary precipitants.** Data from a prospective cohort of OTC patients was characterized for interim HA events. (A) Proportion of interim HA events (plasma ammonia >100 μmol/L) due to infectious and dietary precipitants. (B) Change from baseline ammonia. (C) Hospitalization rate due to non-infectious and infectious precipitants. (D) Length of stay (LOS) for non-infectious and infectious precipitants. (E) Use of IV ammonia scavengers for hospitalized non-infectious and infectious precipitants. (C,E) ‘H’ lines indicate *P*<0.05.

The longitudinal database of the UCDC of the Rare Disease Clinical Research Network gathers prospective data on the clinical characteristics of this unique patient population, which can help provide insight into common HA triggers associated with these disorders. Given the anecdotal clinical evidence that patients with UCD experience more severe HA in the setting of intercurrent illness (Singh et al., 2005), the longitudinal database of the UCDC was queried for interim events in hospitalized OTC patients between 1 March 2005 and 7 August 2012. Infectious etiologies of HA were scored when there was clinical and laboratory evidence of viral and bacterial infections. Dietary etiologies included non-adherence and changes in protein and calories. Overall, 57 OTC patients had a reported 148 HA events. Confirmed infectious etiologies accounted for 39/148 (26%) HA events, whereas dietary perturbations accounted for 28/148 (19%) HA events (Fig. 1A). The remaining etiologies (55%) occurred in lower frequencies individually and were varied: stress, medication changes, menses and unknown causes. Because patients with UCD can have elevated plasma ammonia at baseline, we determined changes in plasma ammonia from baseline during infectious and dietary precipitants. Baseline ammonias were calculated as an average of three previous plasma ammonia levels from well clinic visits. Changes in plasma ammonia from baseline were not significantly different between infectious and dietary precipitants (172±100 versus 147±88 μM, *P*=0.34, Fig. 1B). A single individual with elevated baseline ammonia experienced a decrease from baseline during a dietary precipitant. To examine whether increased morbidity was associated with infection, we next examined hospitalization rate, length of hospital stay (LOS) and intravenous (IV) ammonia scavenger use in a dichotomous fashion for infectious versus non-infectious etiologies. For OTC patients, the hospitalization rate (Fig. 1C) for infection was 22% higher than without infection (*P*=0.04). The average LOS was increased by 1.7 days with infection (Fig. 1D), although this finding was not significant (*P*=0.20). With symptomatic HA, OTC patients typically require the administration of IV ammonia scavengers. When IV ammonia scavengers were used as a surrogate for morbidity during hospitalization (Fig. 1E), the rate of inpatient utilization during acute HA due to infectious (31/39 events; 79%) versus non-infectious (56/102; 55%) precipitants was significantly increased (*P*<0.0001). Overall, these data indicate that infectious precipitants of acute HA in UCD have unique clinical parameters, raising the possibility of distinct pathophysiological mechanisms of ammonia metabolism during infection.

### Development of a model of acute HA due to viral illness

**Fig. 2.**
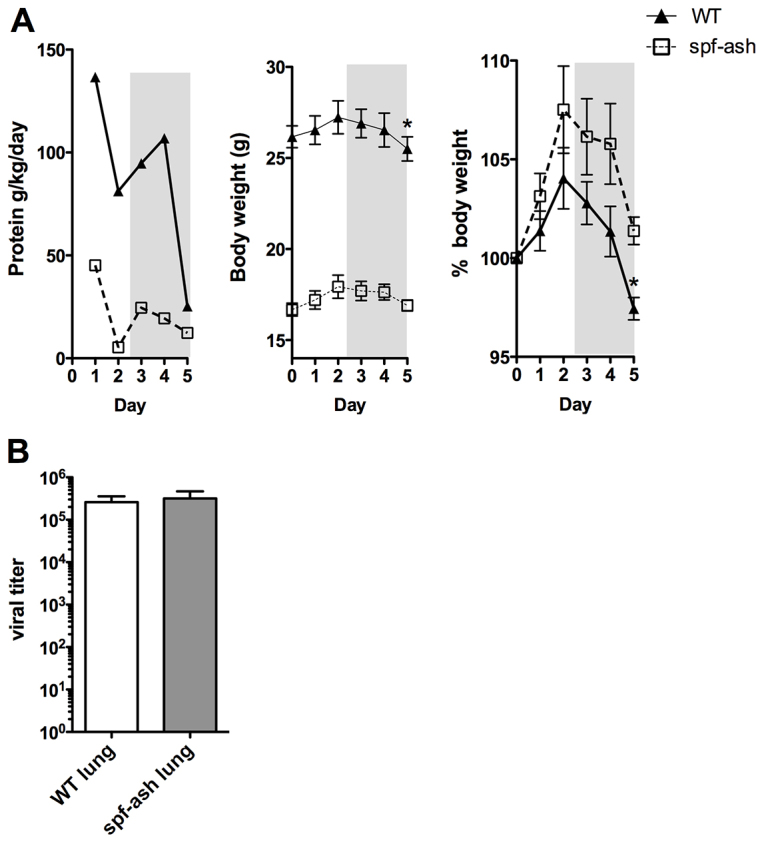
**Infectious parameters in WT and *spf-ash* mice.** Mice infected with PR8 influenza were euthanized at Day 5 and lungs were harvested. (A) Food intake and weight loss (*n*=5/group). Shaded areas indicate appearance of sickness behaviors. For food intake and weight, *P≤0.05. (B) Viral titers in homogenized lungs on Day 5 (*n*=5–6/group).

Because of the distinct epidemiological parameters of infection in UCD (Fig. 1), we next sought to develop an *in vivo* model of acute HA due to infection. Two well-characterized experimental systems – the *spf-ash* mouse and the mouse-adapted PR8 influenza virus – were combined to create a murine model of viral infection in the setting of a UCD. *Spf-ash* and WT mice were infected on Day 0 using an infection aerosolization apparatus and euthanized on Day 5 (supplementary material Fig. S2A,B). *spf-ash* and WT challenged with PR8 were observed to have ruffled fur and decreased social and grooming behaviors beginning at Day 2–3 of the infection protocol (supplementary material Fig. S2A, gray shading). Protein intake, assessed by weighing the food daily, was recorded during the course of infection and expressed on a g/kg body weight/day basis (Fig. 2A). *spf-ash* mice had a lower body weight and protein intake (Day 1, 45.2 g/kg/day) compared with WT (136.6 g/kg/day) and had much lower body weights at baseline, typically weighing 20% less than sex- and age-matched littermates. Both groups decreased their protein intake over the course of the infection, with WT demonstrating a greater decrease (81.6%) versus *spf-ash* (72.8%) by Day 5. WT animals also lost a greater percentage of body weight at Day 5, likely reflecting greater body stores at baseline (Fig. 2A); controls were 97.4% of starting body weight, whereas *spf-ash* were 101.3% of starting body weight (*P*=0.01). From Day 2–5, with onset of sickness behaviors, both WT and *spf-ash* lost weight at similar rates.

### *spf-ash* and WT mice have similar lung infectious parameters

Although *spf-ash* and WT animals both displayed physiological and behavioral characteristics suggestive of viral infection, we assessed viral titers and cytokine profiles in the lungs to confirm that infectious parameters were similar between the strains. Viral titers (Fig. 2B) were measured on Day 5 for lung homogenates using serial dilutions and measuring infectivity in Madin-Darby canine kidney (MDCK) epithelial cells. Whole lungs from infected WT and *spf-ash* mice displayed no differences in viral titers on Day 5 of infection (*P*=0.77). Lung cytokine profiles were determined using a fluorescent-bead-based multiplex assay (Table 1). Both WT and *spf-ash* showed a robust response to viral infection with significant increases in a number of colony-stimulating, chemotactic and inflammatory cytokines [Infection (Inf), *P*<0.05]. No significant genotype differences were seen [Genotype (Gen), *P*>0.05]. With the exception of lower GM-CSF concentrations at Day 5 in *spf-ash* (Inf × Gen, *P*<0.031), all remaining infection × genotype interactions were not statistically significant (Inf × Gen, *P*>0.05). Thus, WT and *spf-ash* displayed similar infectious and inflammatory parameters during PR8 influenza infection.

**Table 1.**
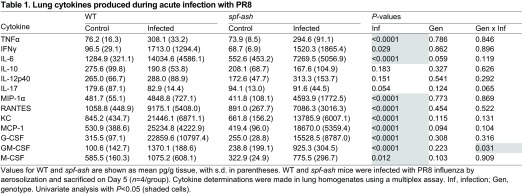
Lung cytokines produced during acute infection with PR8

### Hepatic response during PR8 infection

**Fig. 3.**
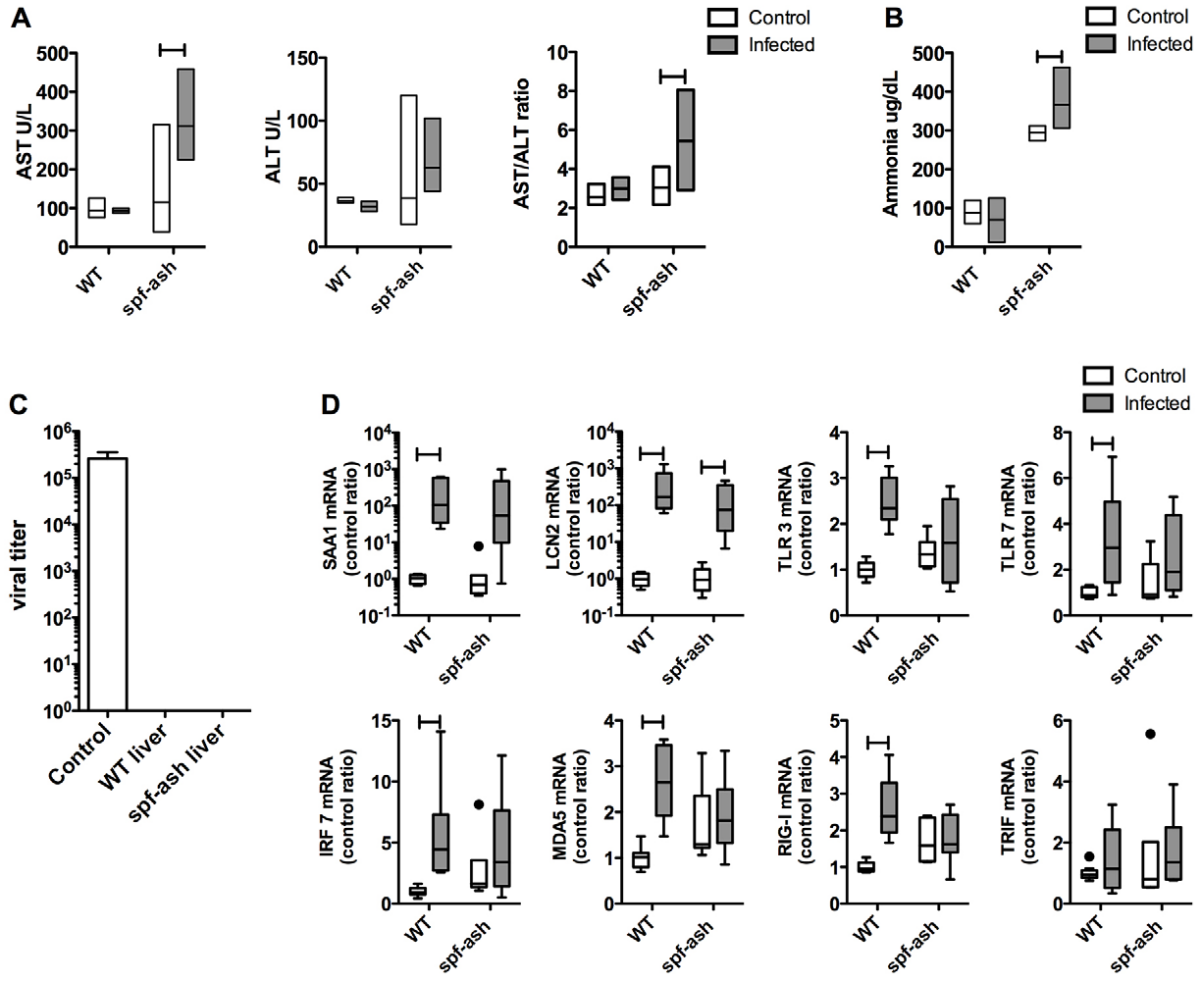
**Hepatic immune response to infection.** Mice infected with PR8 influenza (*n*=5–8/group) were euthanized at Day 5 and tissues were collected by cardiac puncture. (A) Levels of the liver transaminases AST and ALT, and the AST:ALT ratio. (B) Plasma ammonia concentration. (C) Liver viral titer. Control – infected mouse lung tissue. (D) qRT-PCR for liver immune response. SAA1 – serum amyloid A1, LCN2 – lipocalin 2, TLR3 – toll like receptor 3, TLR7 – toll like receptor 7, IRF7 – interferon regulatory factor, MDA5 – melanoma differentiation-associated gene 5, RIG-I – retinoic acid-inducible gene 1, TRIF – TIR-domain-containing adapter-inducing interferon-β. For clinical chemistry floating bar graphs: top=highest value, bottom=lowest value, line=mean. For qRT-PCR, box plots were used. For all, ‘H’ line indicates *P*<0.05. Each experiment was repeated three times.

It is well documented that humans and certain mouse strains can develop elevated serum aspartate aminotransferase (AST) and alanine aminotransferase (ALT) during respiratory viral infection (Polakos et al., 2006). However, whether these signs of hepatic insult translate into alterations in UC function has not been studied. Given the similar parameters of PR8 infection in both WT and *spf-ash*, we performed further biochemical studies to evaluate hepatic metabolic function (Fig. 3). *spf-ash* mice displayed greater hepatocyte sensitivity to infection, with increases in serum AST 3× over control animals (Fig. 3A, *P*=0.007). Serum ALT was on average 2× higher than controls, although this finding was not significant (Fig. 3A, *P*=0.25). Furthermore, the mean AST:ALT ratio in the infected *spf-ash* was 5.4 (Fig. 3A, *P*=0.01), suggesting an exogenous insult as the cause of the hepatitis. Acute metabolic decompensation due to intercurrent illness in patients with UCD is characterized by HA. Consistent with the previous studies in these mice, plasma ammonia was 3 to 4× greater at baseline in *spf-ash* mice compared with WT. However, during PR8 infection, plasma ammonia increased over 100 μg/dl in some animals (*P*=0.04), whereas WT levels remained unchanged from baseline (Fig. 3B). With significant elevations of markers of hepatic damage, liver histology was investigated in infected *spf-ash* and controls. Significant inflammation, necrosis and apoptosis were absent in all the liver samples (supplementary material Fig. S3). Although the increase in liver transaminases and ammonia seen in the plasma were suggestive of Reye syndrome in the *spf-ash* mice, macrovesicular [hematoxylin and eosin (H&E) stain, supplementary material Fig. S3A] and microvesicular (oil red O stain with baking, supplementary material Fig. S3C) steatosis were absent.

Analysis of viral load by qRT-PCR (data not shown) and measurement of viral titer (Fig. 3C) showed that no PR8 virus was present in *spf-ash* livers. Despite a lack of virus detection in unperfused livers, *spf-ash* livers showed signs of hepatic sensitivity and damage (Fig. 3A). Profiling of the hepatic response to lung PR8 infection in WT B6 mice using mRNA expression arrays showed significant increases in the acute phase response and antiviral response pathways (our unpublished data). To examine the hepatic response to PR8 infection, we profiled a select panel of expressed acute-phase response and antiviral response genes in WT and *spf-ash* mice during infection (Fig. 3D). Both WT and *spf-ash* showed a robust increase in serum amyloid A1 (*SAA1*) and lipocalin 2 (*LCN2*), markers of the acute-phase response (*P*<0.05 for both). However, when profiling the hepatic antiviral immune response, some discrepancies emerged. *spf-ash* showed elevations in *TLR3*, *TLR7*, *MDA5* and *RIG-I* at baseline and, unlike WT, failed to show a clear differentiation in levels of expression with infection. Overall, these data suggest that *spf-ash* have an abnormal hepatic immune response to infection and, in some instances, increased activation of antiviral pathways at baseline.

### CPS1 and OTC enzyme activities are reduced in WT and *spf-ash* during infection

**Fig. 4.**
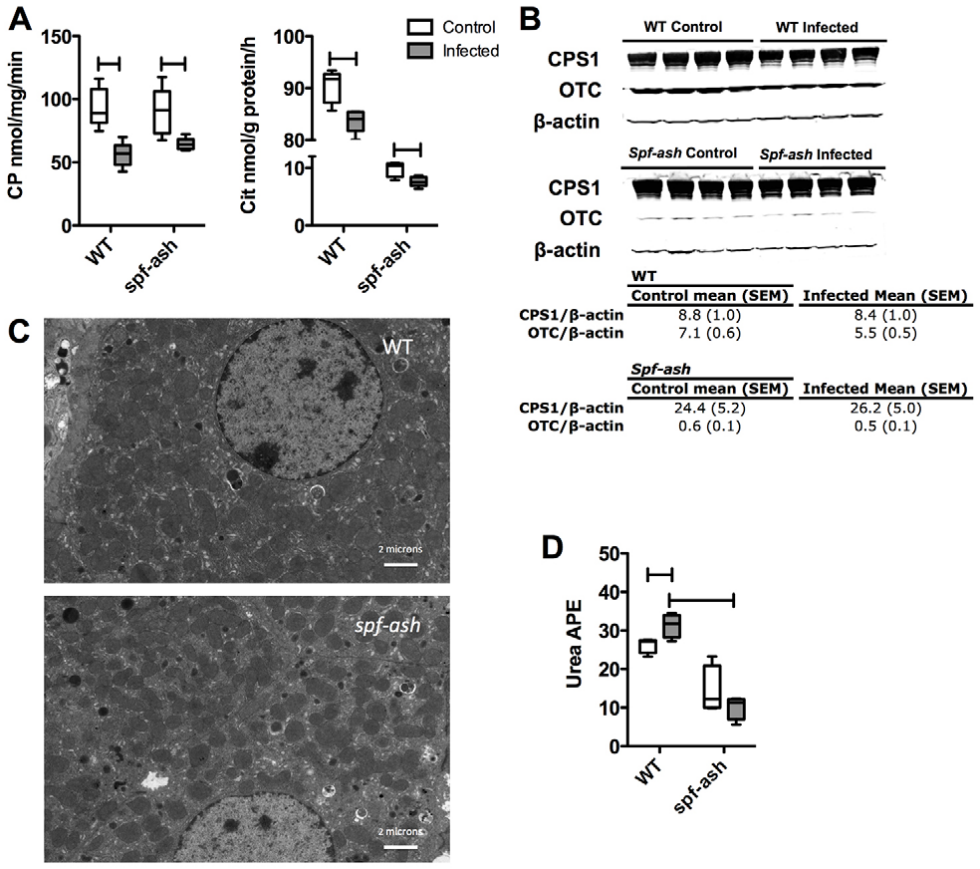
**Hepatic metabolic response during infection.** Mice (*n*=4/group) matched for intake +/− infection with PR8 influenza were euthanized at Day 5 and livers were harvested. (A) CPS1 (CP) and OTC (Cit) enzyme activity. (B) Immunoblot of CPS1 and OTC protein. (C) Electron microscopy of livers on Day 5 of infection. (D) Ureagenesis during infection. For ureagenesis, mice were injected with 4 mmol/kg of ^15^NH_4_Cl and sacrificed 20 minutes after injection (*n*=4/group) (Cunningham et al., 2009). ^15^N-urea enrichment was determined by GC-MS. For all, ‘H’ line indicates *P*<0.05.

In the setting of biochemical abnormalities indicating hepatitis with increased HA, we hypothesized that perturbations in hepatic nitrogen metabolism might be exacerbated during infection. Previous studies have indicated altered mitochondrial UC enzyme function in the setting of influenza infection (Pierson et al., 1976) but did not account for protein intake, which we find varies between WT and *spf-ash* animals. Because the expressions of UC enzymes are responsive to dietary signals (Snodgrass, 2004), all animals were housed individually and matched for protein intake on a g/kg body weight/day basis during the 5 days of infection to correct for protein-intake differences (Fig. 2A). Protein matching resulted in a factorial design (supplementary material Fig. S4A,B). Under this caloric-restriction regimen, weight loss in all experimental groups was similar on Day 5 (supplementary material Fig. S4C). To assess the depression of mitochondrial UC function, CPS1 and OTC activities were measured in liver homogenates (Fig. 4A). During infection, CPS1 activity decreased by 40% (*P*=0.001) in WT and 28% (*P*=0.018) in *spf-ash*, whereas OTC activity decreased by 7% (*P*=0.005) in WT and 21% (*P*=0.015) in *spf-ash* (Fig. 4A). These results suggest that reduction of CPS1 and OTC enzyme activities are part of the normal hepatic physiology of PR8 infection, which might not be tolerated by a compromised UC in *spf-ash*.

To further characterize the mechanism of decreased enzyme activity, CPS1 and OTC were quantified for protein expression differences (Fig. 4B). Surprisingly, CPS1 protein levels were threefold higher in *spf-ash* compared with WT, and infection had no significant effect on CPS1 protein levels in either the WT (*P*=0.787) or *spf-ash* (*P*=0.804) animals. The amount of OTC tended (*P*=0.086) to be reduced in WT littermates during infection, whereas OTC protein levels remained unchanged in the *spf-ash* mice.

Given the increased immunoreactive CPS1 in the *spf-ash* liver extracts, we hypothesized that the hepatocytes had an increase in either protein content or number as a compensation for OTC deficiency. To determine whether there was an increase in mitochondrial number, we examined livers from WT and *spf-ash* by electron microscopy on Day 5 of infection (Fig. 4C). WT and *spf-ash* displayed occasional mitophagy and, on average, similar numbers of mitochondria of normal morphology.

### Altered nitrogen disposal in *spf-ash* during infection

Given the demonstration of mitochondrial UC dysfunction by enzymology, stable isotopic tracing was employed to assess alterations in ureagenesis. Using a standard published protocol, enrichment of plasma ^15^N-urea was determined following an intraperitoneal (IP) injection of a single dose of ^15^N-ammonium chloride (4 mmol/kg body weight; ^15^NH_4_Cl) on Day 5 (Fig. 4D). The end product of ammonia disposal, plasma [^15^N]urea, was enriched in WT mice on Day 5 of infection (*P*=0.03). Conversely, plasma [^15^N]urea enrichment was not only lower in *spf-ash* mice during the uninfected state but there was also a failure to increase incorporation of ^15^NH_3_ into urea during the infected state (*P*<0.01).

Given this failure to increase ureagenesis, we next examined free amino acids in liver homogenates to evaluate UC intermediates (Table 2). *spf-ash* had elevated ornithine (Gen, *P*=0.01), aspartate (Gen, *P*=0.024) and arginine (Gen, *P*<0.01) in the absence of infection. However, a depression of these levels was observed during infection, suggesting a strong genotype × infection interaction (ornithine Gen × Inf, *P*=0.018, aspartate Gen × Inf, *P*=0.015 and arginine Gen × Inf, *P*<0.01). Thus, during infection, perturbations in UCD function can be seen in *spf-ash* at the enzyme and metabolite levels.

**Table 2.**
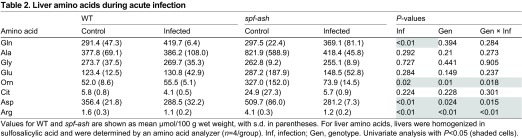
Liver amino acids during acute infection

## DISCUSSION

Life-threatening acute HA is a significant source of morbidity and mortality for patients with UCD, who experience 2.2–2.8 episodes per year on average, depending upon the underlying enzymatic defect (Summar et al., 2008). Prospective analyses of the OTC cohort presented here revealed that infectious precipitants led to increased hospitalization rates (Fig. 1C) and length of hospital stay (Fig. 1D). Consistent with our findings, a recent paper found that inborn errors of metabolism in general are an independent risk factor for hospitalization due to respiratory illnesses such as respiratory syncytial virus (RSV) infection (Kristensen et al., 2012). In addition to increased hospitalization rates and length of stay, infection-associated HA was also accompanied by increased utilization of IV ammonia scavengers (Fig. 1E). Because IV ammonia scavengers are usually reserved for patients with hyperammonemic encephalopathy, this surrogate marker of severity suggests increased morbidity when this patient population is exposed to infectious precipitants. In light of these parameters suggestive of increased morbidity, we aimed to investigate the pathophysiology underlying acute HA induced by infection.

We hypothesized that infection would lead to activation of the immune system with concomitant perturbations in UC function. The factorial design (supplementary material Fig. S4A) adopted in the present study allowed the effects of infection to be assessed in isolation of those related to dietary insufficiency, another common precipitant of HA. In this respect, although dietary insufficiency is present in both conditions, our results clearly indicate that there are distinct metabolic sequelae due to infection.

Although *spf-ash* and WT mice displayed similar markers of lung inflammation (Table 1), the hepatic immune response in *spf-ash* showed some key differences (Fig. 3D). Although virus was not detected in WT livers (Fig. 2B), mRNA elevations in *TLR3*, *TLR7*, *MDA5*, *RIG-I* and *IRF7* were seen in response to infection. Activators of these pathways include the pathogen-associated molecular patterns (PAMPs) dsRNA and ssRNA, which can be seen during viral infection (Seki and Brenner, 2008). In addition to PAMPs, these pathways can be activated in response to dying host cells by damage-associated molecular patterns (DAMPs; e.g. dsRNA, ssRNA, mtDNA). Our findings in WT lead us to suggest that the liver is either reacting to undetectable levels of PR8 virus in the blood and/or liver sinusoidal spaces, or tissue damage from the lung. Interestingly, *spf-ash* display elevations in AST, ALT and markers of innate immune activation in the uninfected state (Fig. 3D). These data suggest that *spf-ash* might be experiencing baseline liver damage with innate immune activation via DAMPs. The baseline immune activation might also account for the hepatic sensitivity seen with infection (AST and ALT, Fig. 3A) and remains to be explored.

A previous study examining the effect of influenza infection on CPS1 and OTC activities in WT B6 mice showed appreciable reductions in CPS1 (12%) and OTC (17%) enzyme activities (Pierson et al., 1976). In the present study, we observed a greater reduction in CPS1 activity likely due to the intake-matching strategy applied (Fig. 4). These findings imply that a reduction in CPS1 and OTC activities is part of the normal physiological response to PR8 infection and might compound the pre-existing OTC deficiency in *spf-ash*. Interestingly, despite lower enzyme activity, immunoreactive CPS1 was actually increased in *spf-ash* mice with normal mitochondrial number and morphology (Fig. 4B,C). Indeed, *spf-ash* liver contains 33% more mitochondrial protein per gram of liver when compared with WT (Cohen et al., 1989). Nonetheless, despite greater CPS1 protein, *spf-ash* CPS1 enzyme activity was 15% lower in *spf-ash* mice. Our results suggest that, even in the uninfected state, a proportion of the CPS1 pool is inactive or subactive in *spf-ash* mice. This *in vitro* reduction in CPS1 activity could be related to altered post-translational modifications such as lysine acylation or acetylation (Duke-Sylvester et al., 2011; Nakagawa et al., 2009), which further suggests an avenue of investigation.

In our model, *spf-ash* mice seem to have lower ureagenesis during dietary restriction (Fig. 4D); however, these findings were not significant. More importantly, unlike WT, *spf-ash* failed to increase ureagenesis during infection (Fig. 4D) and experienced HA (Fig. 3B). This failure of increased ureagenesis was due to a depression in the pool of UC intermediates during infection (Table 2). Although these data reflect a metabolic snapshot of a dynamic process, they are consistent with our additional findings. Overall, the depressions seen in UC intermediates might be due to the observed depression of UC activity (Fig. 4A,D), their incorporation into protein synthesis (Arg and Asp) or activation of other pathways such as polyamine synthesis (Arg and Orn) (Tiao et al., 1995). *spf-ash* mice are able to maintain ureagenesis when given a balanced nitrogen challenge (Marini et al., 2006a; Marini et al., 2006b). When challenged with an unbalanced nitrogen load, profound HA and decreased ureagenesis follows. These results suggest that the prevision of UC intermediates in the balanced nitrogen load can sustain ureagenesis in the setting of a hypomorphic OTC enzyme. As further evidence, supplementation with UC intermediates can also prevent ammonia toxicity after a lethal dose of ammonia (Ben-Ari et al., 2010; Matsuda et al., 1996) or an unbalanced nitrogen load (Marini et al., 2006b). Of particular interest is ornithine. In B6 mice, an IP challenge with ammonium chloride results in elevations in hepatic ornithine within 5 minutes of injection, suggesting its importance in the incorporation of ammonia (Saheki et al., 1997). In *spf-ash* primary hepatocytes, ornithine increased ureagenesis and reduced orotic acid production (Moscioni et al., 2006). In the *spf-ash* mouse, ornithine supplementation restored ureagenesis and mitigated HA during parenteral nitrogen loading (Marini et al., 2006a). Inhibition of ornithine aminotransferase might also be effective (Li et al., 1999). This efficacy of ornithine seems to be related to an increase in OTC and CPS1 activities, and a decrease in carbamoyl phosphate degradation (Ben-Ari et al., 2010; Neill et al., 2009; Seki and Brenner, 2008; Tiao et al., 1995).

In conclusion, we have developed a model of acute metabolic decompensation due to infection in the *spf-ash* mouse. From a therapeutic standpoint, given the depletion of hepatic arginine and ornithine in our model, formal studies on HA and ureagenesis during infection while supplementing these amino acids will provide insight into their efficacy. Overall, this model might serve as a platform for describing biochemical perturbations and the efficacy of treatments during acute metabolic decompensation in UCD due to a common precipitant. In addition, this model system could be adapted to explore acute decompensation due to infection in other types of inborn errors of metabolism such as organic acidemias and fatty-acid-oxidation defects.

## MATERIALS AND METHODS

### Infection with A/PR/8/34 (PR8)

The experiments outlined were performed on B6 × B6EiC3Sn *a*/*A-Otc^spf-ash^*/J (*spf-ash*) and littermate controls (The Jackson Laboratory, Bar Harbor, ME). Mice were housed in a pathogen-free facility, caged individually, had access to a 24% protein mush-based feed, Nutragel (Bio-Serv, Frenchtown, NJ), and autoclaved reverse osmosis water. Mice were kept in a temperature (22±2°C)- and humidity (30–70%)-controlled environment with a 12-hour light cycle. Mouse adapted human influenza virus A/PR/8/34 (PR8) for infection was produced as described previously (Fernandez-Sesma et al., 2006). 4- to 6-week-old *spf-ash* and littermate control mice were exposed to an infective dose of PR8 of 500 TCID_50_ in an aerosolization chamber (Glas-Col, Terre Haute, IN) (Moltedo et al., 2009). Mice were sacrificed on Day 0 and Day 5 of infection by 5% isoflurane inhalation with cervical dislocation. Plasma, serum and tissues were separated from whole blood and stored at −80°C until use. All animal care and procedures were carried out according to the criteria outlined in the ‘Guide for the Care and Use of Laboratory Animals’ prepared by the National Academy of Sciences and published by the National Institutes of Health (NIH publication 86–23 revised 1985) and were authorized by the Animal Care and Use Committees of the National Human Genome Research Institute and the Institutional Animal Care and Use Committee of the Mount Sinai School of Medicine.

### Viral lung titer

Viral titers were determined using a published assay based on the infection of MDCK cells (Oh et al., 2000). The inverse of the dilution at which 50% of the wells showed cytopathic effect was recorded as the 50% tissue culture infectious dose (TCID_50_).

### Lung cytokines

On Day 5 of infection, lungs were removed and immediately homogenized with a TissueRuptor (Qiagen, Valencia, CA) handheld rotor-stator homogenizer in PBS. Cytokine detection in clarified tissue lysates was performed using a mouse cytokine panel (Millipore, Billerica, MA) with detection using a multiplex platform (Luminex, Austin, TX). Cytokine amounts were normalized to lung weight in grams.

### Histology

H&E staining was performed on sectioned paraffin-embedded lung and liver tissue by the Department of Pathology, Mount Sinai Medical Center. Detection of apoptotic cells was performed using the ApopTag Plus Peroxidase *In Situ* Apoptosis Detection Kit (Millipore, Billerica, MA).

### Biochemical studies

Plasma ammonia was determined using a glutamate-dehydrogenase-based assay according to manufacturer instructions (Sigma-Aldrich). Serum aspartate aminotransferase, alanine aminotransferase and urea determination were performed in the Center for Comparative Medicine and Surgery, Mount Sinai School of Medicine. Liver amino acids were quantified by ion-exchange chromatography using a Biochrom 30 Amino Acid Analyzer (Biochrom, Cambridge, UK). Amino acid concentrations were calculated as μmol/100 gram of tissue and expressed as ratios relative to controls.

### Measurement of ureagenesis

Mice were administered a dose of nitrogen as (^15^N)-labeled ammonium chloride according to experiments described previously (Cunningham et al., 2009; Plata-Salamán, 1998). After a 3-hour fast, mice received 4 mmol/kg body weight of ^15^NH_4_Cl (Cambridge Isotope Laboratories, Andover, MA) by IP injection. Heparinized blood was collected by retro orbital bleeding 20 minutes after injection, and the plasma analyzed for the % of [^15^N] isotope enrichment of urea by gas chromatography/mass spectrometry (GC-MS) (Galloway et al., 2000).

### OTC and CPS1 enzyme assays

OTC enzyme activity was measured using a published colorimetric assay that detects the formation of L-citrulline (Pastralandis et al., 1981). CPS1 enzymatic activity was performed using lysates prepared as in the OTC assay according to a published assay (Chan et al., 2009).

### qRT-PCR

Liver tissue was thawed and homogenized in RIPA buffer on ice. DNA and RNA was extracted from homogenized liver tissue or cell pellets using a kit (Qiagen). For RNA, 1 μg was reverse transcribed to cDNA using a modified MMLV-reverse transcriptase (iScript, Bio-Rad, Hercules, CA). Real-time quantitative PCR reactions were carried out in 50 μl using iQ SYBR Green Supermix (Bio-Rad, Hercules, CA) or TaqMan systems (Applied Biosciences, Carlsbad, CA). Reactions were cycled and quantitated with an ABI 7500 Fast Real Time PCR System (Applied Biosystems, Foster City, CA).

### Western blot analysis

For western blot analysis, 30 μg of protein was loaded on 4–20% Tris-glycine polyacrylamide gels. The gels were transferred to polyvinylidene difluoride membrane using the iBlot Dry Blotting System (Life Technologies, Grand Island, NY). The membranes were blocked and probed with primary antibodies according to the manufacturers’ suggested dilutions: CPS1 (Abcam, Cambridge, MA), OTC (Novus Biologicals, Littleton, CO) and β-actin (Sigma-Aldrich, St Louis, MO). Incubation was done with appropriate secondary antibodies. Image analyses were performed using an Odyssey Imager (Li-Cor, Lincoln, NE).

### Electron microscopy

Mouse livers (1 mm^3^) were fixed overnight at 4°C in 2% glutaraldehyde in 0.1 M cacodylate buffer (pH 7.4) and washed with cacodylate buffer three times. The tissues were fixed with 2% OsO_4_ for 2 hours, washed again with 0.1 M cacodylate buffer three times, washed with water and placed in 1% uranyl acetate for 1 hour. The tissues were subsequently serially dehydrated in ethanol and propylene oxide and embedded in EMBed 812 resin (Electron Microscopy Sciences, Hatfield, PA). Thin sections, approx. 80 nm, were obtained by utilizing the Leica ultracut-UCT ultramicrotome (Leica, Deerfield, IL) and placed onto 300 mesh copper grids and stained with saturated uranyl acetate in 50% methanol and then with lead citrate. The grids were viewed in the JEM-1200EXII electron microscope (JEOL Ltd, Tokyo, Japan) at 80 kV and images were recorded on the XR611M, mid mounted, 10.5 Mpixel CCD camera (Advanced Microscopy Techniques Corp., Danvers, MA).

### Statistical analyses

For the Rare Disease Clinical Research Network-sponsored Urea Cycle Disorders Consortium (RDCRN UCDC) longitudinal study, OTC patients who were hospitalized owing to HA events during the course of the study were examined. Because each participant can experience more than one HA event, the generalized estimating equation (GEE) adjusted for age at the HA event was used. Observed frequency (%) for categorized variables and the mean (standard deviation) for continuous variables were reported. *P*-values less than 0.05 were used to indicate statistical significance.
